# When I am on the verge of departing (a poetic advance directive, for my loved ones)

**DOI:** 10.1017/S1478951525000185

**Published:** 2025-02-21

**Authors:** Carlos Eduardo Paiva

**Affiliations:** 1 GPQual - Research Group on Palliative Care and Quality of Life - Barretos Cancer Hospital, Barretos, SP, Brazil; 2 Department of Clinical Oncology, Breast and Gynecology Division, Barretos Cancer Hospital, Barretos, SP, Brazil

When my little vessel drifts into the boundless sea,
Do not anchor me to the shore
Just to capture a shadow of what I was.
Remember my values, not the garments I wore.

When my roots grow weak
And my leaves begin to fall,
Do not drown me with tap water
Or feed me with lifeless care.

When my light fades
And my batteries are spent,
Do not keep me in the workshop,
Futilely searching for the spark that is gone.

Let me sail freely on open waters,
Bearing fruit, offering shade,
Lighting paths for my children,
As long as the heavens permit.
Let me see with my eyes, speak with my voice,
And walk with my own two feet
In garments that are mine alone.

When hope has fled,
Do not cling to the final thread
Just to ease your pain.
The sea, the earth, and the infinite are beautiful,
And when hope fades here,
Another wondrous realm awaits
A new journey just beyond.

When all doors have closed,
Do not shatter my walls in desperation.
I was never meant to be forced.

All I ask is your presence and warmth –
Your hands, your voice, your love.
My music, pizza, ice cream, and soccer on TV.
Time spent with you,
And space to simply be.

If I wish to rest in silence,
Stay near,
Hold my hand,
Let soft music play in the background,
A soothing melody, a prayer from the heart.
Speak the truths I hold dear,
And weep gently, if you must.

